# Comparison of Mast Cell Count in Oral Epithelial Dysplasia and Squamous Cell Carcinoma

**DOI:** 10.30476/dentjods.2024.99652.2164

**Published:** 2024-12-01

**Authors:** Saede Atarbashi-Moghadam, Maral Niazmand, Shahla Vafadar, Sanaz Gholami Toghchi

**Affiliations:** 1 Dept. of Oral and Maxillofacial Pathology, School of Dentistry, Shahid Beheshti University of Medical Sciences, Tehran, Iran; 2 General Dentist, Research Center, Dental School Shahid Beheshti University of Medical Sciences, Tehran, Iran

**Keywords:** Squamous Cell Carcinoma of Head and Neck, Oral Leukoplakia, Mast cells, Inflammation

## Abstract

**Statement of the Problem:**

Squamous cell carcinomas (SCCs) and premalignant disorders such as leukoplakia are common oral cavity lesions. Although these lesions are epithelial in nature, they are also associated with juxta-epithelial chronic inflammation. Mast cells play a significant role in inflammation initiation and propagation

**Purpose:**

Previous studies have yielded conflicting results in this field. Therefore, this research aimed to assess the number of mast cells in oral SCC and dysplastic leukoplakia and explore their possible role in these lesions

**Materials and Method:**

In this retrospective cross-sectional study, sixty-three archival cases, including 22 OSCCs, 28 dysplastic leukoplakias as epithelial dysplasia (ED), and 13 normal oral mucosal tissues, were examined for mast cells, using toluidine blue staining. Hotspot areas were identified at 10× magnification and mast cells were counted in 5 fields at 40× magnification. The average cell numbers were calculated, and the severity of inflammation was scored. Statistical analysis was performed using SPSS statistical software 20, including One-way ANOVA, Two-way ANOVA, paired-t test, and independent t-test. *p* Value < 0.05 was considered significant

**Results:**

Among the 51 pathologic lesions, 54.9% were males and 45.1% were females, with a mean age of 56.34±15.35 years. The most common locations were the tongue and buccal mucosa. The mast cell count was significantly lower in SCC compared to ED (*p*= 0.009). There was no correlation between mast cell count and inflammation score (*p*= 0.345)

**Conclusion:**

In this study, the mast cell count was higher in ED compared to OSCC, suggesting an increase in these cells during the pre-malignant stages. However, the number of mast cells decreased after connective tissue invasion and microenvironmental changes occurred

## Introduction

Oral potentially malignant disorders (OPMDs) encompass a range of asymptomatic clinical manifestations that precede the development of oral cancer. Among these disorders, leukoplakia is particularly associated with a high tendency of dysplasia [ 1 ]. Dysplastic changes in the stratified squamous epithelium are characterized by cellular atypia and the loss of normal maturation and stratification [ 2 ]. However, other factors such as papillary/verrucous architecture, bulky epithelial hyperplasia, epithelial atrophy, and "skip" segments should also be considered when evaluating oral ED, especially in cases when typical cellular features of dysplasia are minimal or absent [ 3 ]. The severity of dysplasia is directly correlated with the likelihood of malignant progression, although non-dysplastic lesions can also transform into malignancy [ 2 ]. SCC is the most common type of oral cancer worldwide [ 4 ]. Despite efforts to detect premalignant lesions and early-stage SCCs, the survival rate of patients has shown limited improvement over the past several decades [ 5 ]. Mast cells are mobile immune cells, derived from the bone marrow and characterized with granule-containing cytoplasm. They are present throughout the body, including all connective tissues and mucosal environments, with a particular abundance in perivascular areas [ 6 ]. Mast cells have been associated with increased mitotic activity, microvascular hyperpermeability, extracellular matrix degradation, angiogenesis, and recruitment of inflammatory cells, including macrophages. The role of mast cells in cancer is still a subject to debate, as their contribution to tumor progression versus their potential antitumor influence remains unclear. However, Gudiseva *et al*. [ 7 ] suggest that mast cells can play both positive and negative roles in tumor growth. 

This study aims to compare the mast cell count in oral ED and SCC and investigate their possible roles in tumor progression. 

## Materials and Method

This retrospective cross-sectional study was approved by the Ethics Committee of Shahid Beheshti University of Medical Sciences (IR.SBM. DRC.REC.1400.163). Archived samples from the Oral Pathology Department at Shahid Beheshti University of Medical Sciences and the Pathology Department of Amir A’lam Hospital were evaluated. Samples diagnosed with dysplasia (clinically diagnosed as leukoplakia) and SCC were selected for analysis. Demographic information, including age, gender, and lesion location, was collected from patients’ records. The histopathologic grade for ED (mild and moderate/severe) and SCC (well and moderately differentiated) were recorded, and the degree of inflammation was assessed for each case. A total of 28 ED (12 mild, 16 moderate/severe), 22 SCC cases (19 well and 3 moderately differentiated), and 13 samples of normal oral mucosa, were chosen.

From each selected paraffin block, a 5-µm thick section was obtained. Mast cell detection was performed by staining the sections with 1% toluidine blue. Mast cell granules appeared as red-purple masses in the blue background of cells. The selected areas for mast cell counting were the connective tissue subjacent to the epithelium in oral ED and normal mucosa. Mast cells were counted in 5 high-power fields (HPF), specifically in the areas with the highest mast cell density (hotspots), by two oral pathologists using a 5-eyed optical microscope (Nikon). The number of mast cells in each field was counted, and the total count was considered as the mast cell count (MCC). The intensity of inflammatory cell infiltration was assessed using a scale ranging from 0 (absence) to 3 (severe). The data were analyzed using SPSS statistical software 20, employing One-way ANOVA, Two-way ANOVA, paired-t test, and independent t-test. Comparisons between groups were evaluated using Two-way ANOVA analysis. In SCC samples, peri- and intra-tumoral areas were compared by paired-t test. p <0.05 was considered significant.

## Results

**Figure 1:**
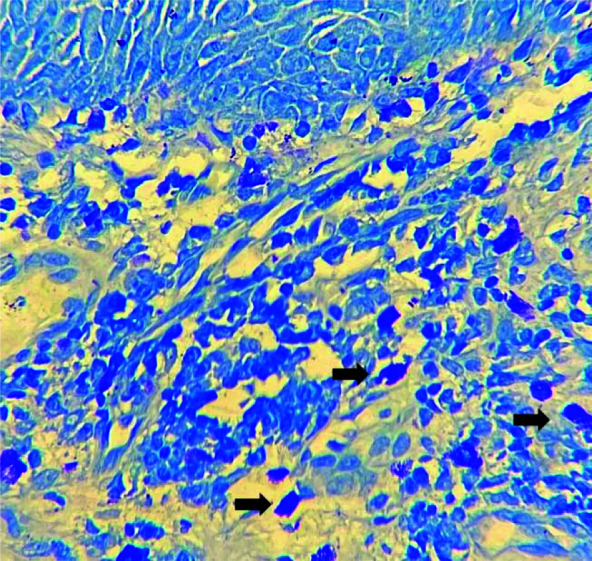
Mast cells (black arrows) in the lamina propria in severe dysplasia in the form of large oval cells with basophilic granules (Toluidine blue ×400)

The demographic data of patients with ED and SCC are described in Table 1. Mast cells were observed as oval or short spindled cells with basophilic cytoplasmic granules, and some showed degranulation (Figures 1). A comparison of intra-tumoral and peri-tumoral MCC in SCC using paired-t test revealed no significant difference (Table 2). Therefore, the means of intra-tumoral and extra-tumoral MCCs in SCCs were used. According to the One-way ANOVA, the MCC was significantly higher in EDs than in OSCCs (*p*= 0.009) (Table 3).

**Table 1:** Demographic data of oral epithelial dysplasia (ED) and oral squamous cell carcinoma (SCC)

		OSCC	ED	Total
**Mean age**		60±16.8	57±15.5	56.34±15.35
Gender	F	8(34.78%)	15(53.57%)	23(45.09%)
	M	15(65.22%)	13(46.43%)	28(54.91%)
Location	Gingiva	7(30.43%)	2(7.14%)	9(17.65%)
	Buccal mucosa	3(13.04%)	11(39.29%)	14(27.45%)
	Tongue	11(47.83%)	11(39.29%)	22(43.14%)
	Others	2(8.7%)	3(10.71%)	5(9.8%)

**Figure 2:**
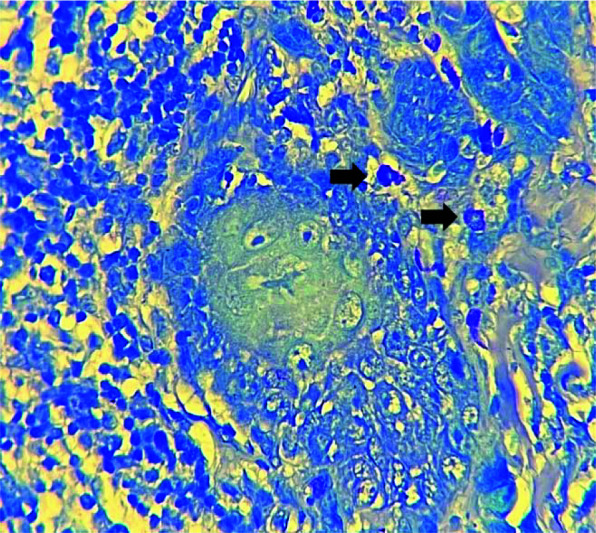
Mast cells (black arrows) in intra-tumoral area of oral squamous cell carcinoma (SCC) (Toluidine blue ×400)

**Figure 3:**
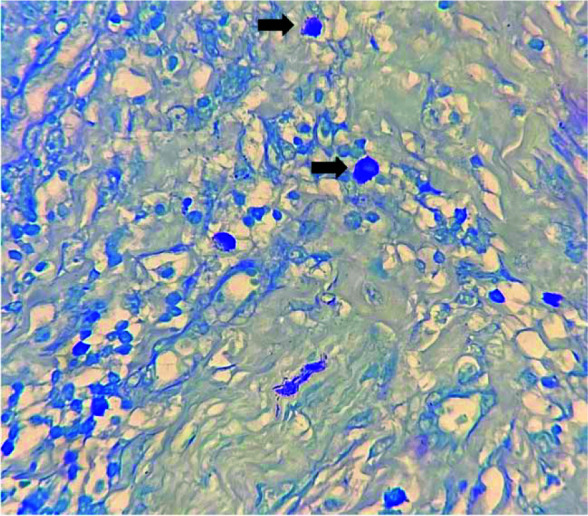
Mast cells (black arrows) in peri-tumoral area of oral squamous cell carcinoma (SCC) (Toluidine blue ×400)

**Table 2:** Comparison between intra- and peri-tumoral mast cell count (MCC)

MCC	Mean	SD	*p* Value
OSCC			
Intra-tumoral	5.41	2.47	0.432
Peri-tumoral	6.09	3.56	

**Table 3:** Comparison between mast cell count (MCC) in oral epithelial dysplasia (ED) and oral squamous cell carcinoma (SCC)

MCC	Mean	SD
Diagnosis		
OSCC	4.71	1.96
ED	6.79	2.8

By implementing the Independent-t test, significant differences were found between well-differentiated and moderately-differentiated SCCs. However, there was no significant difference between mild and moderate/ severe EDs (*p*= 0.026 and 0.533, respectively) (Table 4). Two-way ANOVA showed no significant correlation between MCC, clinical diagnosis, age (*p*= 0.398), gender (*p*= 0.952), and inflammation intensity (*p*= 0.244).

**Table 4:** Comparison of mast cell count (MCC) between grades of oral epithelial dysplasia (ED) and oral squamous cell carcinoma (SCC)

Microscopic data	Grade	MCC		
Diagnosis		Mean	SD	*p* Value
SCC	Well-differentiated	4.35	1.56	0.026
	Moderately-differentiated	6.98	3.07	
ED	Mild	6.4	2.48	0.533
	Moderate / severe	7.11	3.17	

## Discussion

In the present study, the MCC in oral ED was higher than normal mucosa and OSCC, which is consistent with several previous studies in this field [ 8 - 10 ]. Mast cells have been reported to play a role in various OPMDs such as oral leukoplakia, oral lichen planus, and oral submucosal fibrosis. For instance, interleukin-1 released from degranulated mast cells is associated with epithelial proliferation in oral leukoplakia. Histamine, on the other hand, increases mucosal permeability, facilitating the access of stimuli to the underlying connective tissue, and increasing the likelihood of ED [ 8 - 10 ]. In oral lichen planus, activation of CD8 cells leads to mast cell degranulation through its cytotoxic effect. Furthermore, degranulated mast cells release TNF-alpha, which leads to the breakdown of basement membrane integrity through the release of matrix metalloproteinase-9 [ 8 ]. In the presence of a carcinogenic environment, continuous oncogenic signaling leads to the transformation of normal cells into OPMDs and further into OSCC [ 7 ].

In current study as the lesions progressed from premalignant to malignant stages, the MCC significantly decreased. Similar findings were reported by Singh *et al*. [ 9 ] and Shrestha *et al*. [ 8 ]. Oliviera *et al*. [ 11 ] suggested that when the tumor microenvironment stabilizes, the migration of mast cells is more likely to fail at the tumor site due to their protective role. It has also been suggested that the failure of mast cell infiltration could be due to a decrease in chemotactic factors and a reduction in the activation pathway of c-kit [ 12 ].

In contrast to our findings, Iamaroon *et al*. [ 13 ] and Michailidou *et al*. [ 14 ] found a significant increase in the number of mast cells in OSCC cases compared to OPMDs. These findings are possibly related to the angiogenic switch that occurs in the early stages of malignant transformation and emphasize the role of mast cells in the progression from normal to dysplastic tissues, leading to the development of OSCCs.

A dual role has been proposed for mast cells in the pathogenesis of OSCCs. Some believe that mast cells contribute to tumor progression through angiogenesis and neovascularization. Conversely, others support the cytotoxic function of mast cells, which suppresses tumor growth potential [ 9 , 11 , 15 ]. It is speculated that in the initial stages of tumorigenesis, mast cells have a cytotoxic effect. However, as a neoplasm forms, the altered microenvironment suppresses the cytotoxic effect of mast cells and promotes tumor growth by increasing angiogenic potential. This possible mechanism is supported by the finding that the cytotoxic effect of mast cells is actively related to a mast cell-to-tumor cell ratio greater than 20 to 1, and this effect is reversed when the ratio changes from 10 to 1 to 1 to 100 [ 15 - 16 ]. 

In a study conducted by Jafari *et al*. [ 5 ], the peri-tumoral and intra-tumoral areas of OSCC were separately evaluated, and the peripheral part showed a higher MCC, which is similar to our findings although it was not statistically significant. The accumulation of mast cells at the tumor margins has been reported by some studies [ 17 - 18 ]. The presence of mast cells in the tumor margins may be attributed to higher vascular density in that area. Chymase in mast cells has been associated with matrix degeneration, angiogenesis, and tumor growth [ 19 ].

In present study, the MCC was higher in advanced grades of ED, although the difference was not statistically significant. Similar findings were reported by Pujari *et al*. [ 20 ] and Santana *et al*. [ 21 ] regarding an increase in MCC in advanced stages of ED and oral submucosal fibrosis.

Mast cells play a crucial role in the development and progression of inflammation from acute to chronic in the oral mucosa. However, in the current study, no correlation was found between the MCC and the severity of inflammation. Farahani *et al*. [ 6 ] reported a significant relationship between MCC and inflammation intensity in irritation fibroma and peripheral giant cell granuloma in their study on oral reactive lesions. Conversely, in a study on odontogenic cysts, no significant correlation was observed between inflammation and the presence of mast cells [ 22 ]. 

In summary, there have been conflicting results regarding the MCC in oral premalignant and malignant lesions, which may be attributed to the type of staining method used. Apart from Toluidine Blue, various staining methods including Giemsa, Alcian Blue, and immunohistochemical staining using anti-tryptase antibodies are employed for mast cell identification, which may yield different results [ 6 ]. Shrestha *et al*. [ 8 ] mentioned that several diagnostic and prognostic immunohistochemical markers are available for OPMDs and OSCCs. However, due to economic constraints and the requirement for advanced laboratory setups for molecular studies, patients with limited financial resources may find it difficult to afford the cost of these methods, making histochemical staining a potential substitute in certain cases.

Declaration of Artificial Intelligence-assisted technologies in the writing process

Authors used ChatGPT to edit this article to enhance clarity and diminish the flaws of the articles. The content has been reviewed and edited by authors after using this tool and authors take full responsibility for this publication. 

## Conclusion

In conclusion, our study indicates that the MCC in ED is higher than that in OSCC, suggesting an increase in mast cell presence during the premalignant stages. However, with the invasion of the connective tissue and the change of the microenvironment, the number of mast cells is reduced.
